# Outcomes of children involved with child protection in Manitoba, Canada: a demonstration project for working in partnership across government, community organizations, and academia.

**DOI:** 10.23889/ijpds.v7i3.1941

**Published:** 2022-08-25

**Authors:** Marni Brownell, Stephanie Sinclair, Nathan Nickel, Marlyn Bennett, Hygiea Casiano, Anita Durksen, Elizabeth Decaire, Lisa Flaten, Kayla Frank, Mikayla Hunter, Nora Murdock, Chris Nash, Jamie Pfau, Heather Prior, Jay Rodgers, Colette Scatliff, Scott Sinclair, Anna Slavina, Randy Walld

**Affiliations:** 1 University of Manitoba; 2 First Nations Health and Social Secretariat of Manitoba; 3 Manitoba Centre for Health Policy; 4 First Nations Family Advocate Office, Assembly of Manitoba Chiefs; 5 Manitoba First Nations Education Resource Centre; 6 Manitoba Families, Government of Manitoba; 7 General Child and Family Services Authority; 8 Central Services, Government of Manitoba

## Objectives

SPECTRUM is a unique multi-disciplinary, cross-sector collaborative research partnership between academics, government, and community organizations in Manitoba working together to address complex social problems. In our first research project, partners identified the need for quantitative evidence around outcomes of children involved with Child Protection Services (CPS), using linked administrative data.

## Approach

From the SPECTRUM partnership, a research team with CPS expertise was established, including government policy-makers, community organizations representing First Nations families, and academics from multiple disciplines. The research is guided by an Advisory Circle of First Nations Knowledge Keepers. Linking health, education, CPS, and justice data we developed a matched cohort identifying children involved with CPS (2007-2018) for whom there was discretion in the decision to 1) place them in out-of-home care (n=19,718), or 2) keep them in their family home while providing services (n=28,154). Instrumental Variable analysis, with CPS agency as the instrument, will be used to compare outcomes.

## Results

Following the trajectories of these two groups of CPS-involved children over time, we will compare their mental and physical health, educational achievement, and justice system involvement while accounting for individual-level (e.g., age, sex, chronic health conditions) and family-level (e.g., family income, maternal mental health, number of siblings) factors that may contribute to these outcomes. Preliminary findings will be workshopped with the SPECTRUM partnership to facilitate discussions on framing the evidence for policy makers. The SPECTRUM policy team will then prepare policy recommendations for government to consider. The Advisory Circle and a youth advisory squad will facilitate contextualizing and mobilizing findings. Actions taken by government in response to material provided will be monitored and will inform the development of subsequent research projects conducted by SPECTRUM.

## Conclusion

Government and community stakeholder involvement throughout bolsters the likelihood of evidence translating into program and policy changes. This is not a situation where academics are telling government how to do their jobs – this is government, community organizations, and academics working together with the shared goal of better outcomes for children.

